# Investigation of Cognitive Distortions in Panic Disorder, Generalized Anxiety Disorder and Social Anxiety Disorder

**DOI:** 10.3390/jcm12196351

**Published:** 2023-10-03

**Authors:** İlker Özdemir, Erkan Kuru

**Affiliations:** 1 Department of Psychiatry, Faculty of Medicine, Giresun University, Giresun 28200, Turkey; 2 Private Practice, Psychiatry, Ankara 06510, Turkey; erkankuru83@gmail.com

**Keywords:** cognitive distortions, anxiety disorders, cognitive behavioral therapy

## Abstract

The aim of this study was to determine the main cognitive distortions observed in panic disorder (PD), generalized anxiety disorder (GAD) and social anxiety disorder (SAD) and to investigate the impact of cognitive distortions on diagnoses, depression levels, disorder type and severity of anxiety. This study consisted of 150 clinical (50 PD, 50 GAD, 50 SAD) and 91 healthy control participants. A sociodemographic data form, the Beck Depression Inventory (BDI), the Dysfunctional Attitudes Scale (DAS), the Cognitive Distortions Scale (CDS) and the State-Trait Anxiety Inventory (STAI) scales were administered to all participants. It was found that cognitive distortions were higher in individuals with PD, GAD and SAD. The PD, SAD and GAD groups were similar for “catastrophizing”, “mindreading”, “all or nothing thinking”, “overgeneralization”, “should statements” and “emotional reasoning”. “Personalization”, “labeling” and “minimizing or disqualifying the positive” were observed at a higher severity in the SAD group compared to the PD group, and “mental filter” was observed at a higher severity in the GAD group compared to the PD group. Our findings emphasize the need to address cognitive distortions in PD, GAD and SAD treatment. The evaluation of cognitive distortions specific to anxiety disorders is significant in guiding therapy goals and pioneering new research.

## 1. Introduction

According to cognitive theory, the basis of mental disorders is assumed to be dysfunctional evaluations or beliefs of individuals in processing external events/situations or inner stimuli [1].

Dysfunctional beliefs in the cognitive domain shape thoughts and lead to a number of cognitive outcomes called “cognitive distortions”, which are characteristic of various psychopathologies [1,2,3]. The aspects of cognitive distortions that lead to psychopathology are that they are exaggerated, too frequent, systematic and inappropriate [4]. Thus, cognitive distortions affect every aspect of an individual’s life and the way they evaluate themselves, their current experiences and their future [5,6]. Types of cognitive distortions include arbitrary inference, mental filter, overgeneralization, minimizing or disqualifying the positive, all-or-nothing thinking, personalization, catastrophizing, should statements, mindreading, emotional reasoning and labeling [7].

In the cognitive model of anxiety disorders, information is biasedly processed through a cognitive schema or belief (either a non-existent danger is evaluated as existing or is misinterpreted as a higher risk than it actually is). The active cognitions of overestimating the possible risk and inadequately evaluating coping skills are at the basis of anxiety disorders [2]. Stress and coping theory argues that when faced with a stressor, perceiving and evaluating stress is key to the stress response [8]. While a successful response produces positive results, unsuccessful responses cause stressors to be perceived as extremely unattractive and uncontrollable, and increase distress [9].

It is emphasized that the key cognition in panic disorder (PD) is catastrophizing [1]. Catastrophic interpretation of ordinary physical symptoms leads to increased autonomic activity and further exacerbation of symptoms. Catastrophic interpretations are further strengthened by maladaptive behaviors such as avoidance and safety-providing behaviors exhibited due to cognitive distortions [1]. Bandura emphasized that in addition to catastrophic cognition, low self-efficacy cognition is one of the main factors in the development of PD [10].

In the formation and maintenance of generalized anxiety disorder (GAD), the cognitive model emphasizes the presence of cognitive distortions such as catastrophizing, labeling, generalization of danger to other stimuli, excessive focus on negative outcomes and intolerance of uncertainty [1,11]. In Barlow’s GAD model, hyper-arousability and anxiety symptoms are focused on. Distorted cognitions of the inability to control and weakness are dominant due to the weakness in coping skills and the perceived lack of support systems [12].

Individuals with social anxiety disorder (SAD) possess dysfunctional attitudes and cognitive distortions about their own behavior and the way others judge these behaviors [13]. It is emphasized that individuals with SAD are more likely to catastrophize for negative events than other anxiety disorders [14].

Regarding treatment, little is known about whether certain types of cognitive distortions are more associated with different clinical presentations. The same is true about whether certain types of cognitive restructuring would be more effective for different types of cognitive distortions [15]. If some types of cognitive distortions can be related to a specific anxiety disorder, it would suggest that prevention and early intervention initiatives may be further improved by directly targeting these prominent cognitive distortions [16].

Dysfunctional attitudes show high parallelism with cognitive distortions and interact with each other [17]. It is known that dysfunctional attitudes play an important role in triggering and intensifying anxiety disorders. This cognitive feature serves to greatly influence the development of exaggerated worry, anxiety and avoidance behaviors, which are often considered hallmark symptoms of anxiety disorders [18].

It has been emphasized that cognitive distortions are positively correlated with depression symptoms and severity [19,20]. Having distorted cognitions about oneself, the environment and the future affects the emotional response to stressors. In this context, cognitive distortions are highly related to depression symptomatology.

This study aimed to evaluate the cognitive content of PD, GAD and SAD, which are among the anxiety disorders with the highest numbers of treatment-seeking and clinical admissions, and to compare them with a healthy control group. The study also aimed to determine the main cognitive distortions observed in PD, GAD and SAD and whether diagnoses, depression and anxiety levels and dysfunctional attitudes have an effect on these cognitive distortions. One of the hypotheses of the study is that SAD, GAD and PD will differ from each other and from the healthy group in terms of distorted cognitions. Cognitive distortions are known to exhibit positive correlations with anxiety and dysfunctional attitudes [17]. However, another hypothesis is that the strength of this correlation may vary based on diagnoses (GAD, SAD and PD) once the influence of severity of depressive symptoms is removed.

## 2. Methodology

### 2.1. Study Plan

The sample of the study consisted of 241 voluntary participants, including 50 individuals with PD, 50 individuals with GAD and 50 individuals with SAD, and 91 healthy individuals, selected randomly using the non-probability sampling method, who applied to psychiatry outpatient clinics between January 2021 and December 2021. The participants of the healthy control group were selected from various professions (e.g., medical school students, clinical psychology students, nursing students, nurses, physicians and social workers) working or training at the hospital. In this cross-sectional study, a diagnostic assessment was conducted using the SCID-I (structured clinical interview for DSM-5) administered by clinicians. Informed consent was obtained from all participants.

The inclusion criteria were determined as being between the ages of 18 and 65 years and having adequate literacy skills. The exclusion criteria were having a neurodevelopmental disorder, having a past history of a psychiatric diagnosis and treatment and having a current additional psychiatric diagnosis. Comorbidity with other anxiety disorders was excluded. Participants with anxiety disorders who had not yet received treatment and who were admitted for treatment for the first time were included in the study.

### 2.2. Data Collection Tools

Sociodemographic Data Form: This form was prepared by the researchers to assess the demographic characteristics of the participants such as age, gender, marital status and educational status, and clinical characteristics such as presence of additional medical illness, presence of mental disorders in the family, alcohol and tobacco use and history of suicide attempts.

Structured Clinical Interview for DSM-5 (SCID-I): The clinical interview form is used in the investigation of mental disorders according to the Diagnostic and Statistical Manual of Mental Disorders, Fifth Edition (DSM-5) [21]. The Turkish adaptation of the form was made by Elbir et al. [22]. For the Turkish adaptation of the scale, the percentage of agreement for all diagnoses was 97.2% and the kappa coefficient was 0.74. The kappa coefficient ranged between 0.65 and 1.00 and all were statistically significant.

Dysfunctional Attitudes Scale (DAS): This Likert-type scale was developed by selecting 17 items from the original form [7]. The scale has two sub-dimensions, namely “Perfectionism/Performance evaluation (P)” and “Dependency (D)”. The Turkish adaptation of the scale was completed by Şahin and Şahin [23]. In the Turkish adaptation of the scale, the split-half reliability was found to be r = 0.72. The Cronbach’s alpha and the mean item–total correlation were 0.79 and 0.34, respectively.

Cognitive Distortions Scale (CDS): This Likert-type scale aims to measure 10 cognitive distortions (mindreading, catastrophizing, all-or-nothing thinking, emotional reasoning, labeling, mental filter, overgeneralization, personalization, should statements, minimizing or disqualifying the positive) in two sub-dimensions, namely interpersonal (IP) and personal achievement (PA), by scoring between 1 and 7 points [24]. The scale was adapted for use in Turkey by Özdel et al. [25]. In the Turkish adaptation of the scale, factor analyses supported a one-factor model. It has been shown to be valid and reliable with excellent internal consistency between individuals with and without depression (Cronbach’s alpha values were 0.91 and 0.93, respectively).

State-Trait Anxiety Inventory (STAI): This scale was developed to evaluate the state and trait anxiety experienced by an individual depending on the current situation [26]. The scale was adapted for use in Turkey by Oner and le Compte and has been demonstrated to be valid and reliable (Cronbach’s alpha value was 0.94) [27].

Beck Depression Inventory (BDI): This self-reporting scale contains 21 items, designed to evaluate emotional, cognitive, somatic and motivational symptoms observed in depression [28]. The scale was adapted for use in Turkey by Hisli and has been demonstrated to be valid and reliable (Cronbach’s alpha value was 0.80) [29].

### 2.3. Data Analysis

The SPSS (Statistical Package for the Social Sciences) 26 software program for Windows (SPSS Inc., Chicago, IL, USA) was used to analyze the data obtained in the study. Categorical variables were expressed as number and percentage, and continuous variables were expressed as mean ± standard deviation. The conformity of the continuous variables to a normal distribution was evaluated according to whether the skewness and kurtosis values were between −1.5 and +1.5 [30]. The Chi-square test was used to evaluate whether categorical variables from sociodemographic characteristics were similar between groups. The one-way ANOVA test was conducted to compare quantitative data with normal distribution between independent multiple groups. It was used to compare sociodemographic characteristics such as age and years of education and BDI, DAS, STAI and CDS scores between groups (PD, GAD, SAD and control groups). In case of statistically significant differences between the groups, the Bonferroni test was applied as a post hoc analysis to compare the groups with each other. The Kruskal–Wallis H test was used to compare non-parametric data between multiple groups, and the Mann–Whitney U Test was used for the post hoc analysis of the groups. The level of relationship between the scales applied to the participants was evaluated using Pearson’s correlation analysis. Significance in statistical analysis was evaluated as *p* < 0.05.

## 3. Results

### 3.1. Sociodemographic Characteristics

When the participants were compared according to their sociodemographic characteristics, it was observed that the groups had similar characteristics in terms of age, duration of education, marital status, presence of additional medical illness, smoking and alcohol use (Table 1).

### 3.2. Clinical Scale Scores

When the groups were compared according to clinical variables, they differed significantly from each other for all the scales (Table 2).

When the PD group was compared with the GAD group in the post hoc analyses, it was observed that the groups showed similar scores in all the scales (Table 3).

When the PD and SAD groups were compared, it was observed that they were similar in terms of the STAI Trait and CDS IP scores, the PD group showed high scores in terms of the STAI State scales, and the SAD group showed high scores in terms of the BDI, DAS and CDS PA scales (Table 3).

When the GAD and SAD groups were compared, it was observed that the GAD group had higher scale scores in the STAI Trait and STAI State scales, and the SAD group had higher scale scores in the DAS Total and P scales (Table 3).

The PD group had higher scores than the control group in all scale scores except for the DAS Total and P scales. The GAD group had higher scores than the control group in all scale scores except for the DAS P scale. The SAD group had higher scores than the control group in all scales (Table 3).

### 3.3. Cognitive Distortions

When the groups were compared according to cognitive distortions, they differed significantly from each other for all cognitive distortions (Table 4).

The PD, SAD and GAD groups were similar for “catastrophizing”, “mindreading”, “all or nothing thinking”, “overgeneralization”, “should statements” and “emotional reasoning” cognitive distortions. “Personalization”, “labeling” and “minimizing or disqualifying the positive” cognitive distortions were observed at a higher severity in the SAD group compared to the PD group, and “mental filter” cognitive distortion was observed at a higher severity in the GAD group compared to the PD group (Table 5).

### 3.4. Correlations of Cognitive Distortions

The correlations of the cognitive distortions with the STAI and DAS scale scores were examined in each of the PD, GAD and SAD groups. In the PD group, it was observed that while the CDS-Total, CDS-IP and CDS-PA scores were correlated with all the scales, their relationship with the STAI-State and STAI-Trait scores vanished or their strength weakened after the partial correlation analysis in the control of the depressive symptom severity, while the relationship with the DAS scores was preserved (Table 6).

In the GAD group, while the CDS-Total, CDS-IP and CDS-PA scores correlated with all the scales, after the partial correlation analysis in the control of depressive symptom severity, it was observed that the relationships with the STAI-State and STAI-Trait scores vanished or weakened, and the relationship between the DAS and CDS-PA scores disappeared. The relationship between the CDS-Total, CDS-IP and DAS scores was preserved (Table 6).

In the SAD group, while the CDS-Total, CDS-IP and CDS-PA scores correlated with all the scales except for STAI-State, after the partial correlation analysis in the control of the depressive symptom severity, it was observed that the relationships with the STAI-Trait and DAS-D scores disappeared, while the relationships with the DAS-Total and DAS-P scores were preserved (Table 6).

## 4. Discussion

In the present study, we aimed to investigate cognitive distortions in anxiety disorders and the factors that may affect them. Individuals diagnosed with PD, GAD and SAD were compared with each other and with healthy individuals.

In this study, all cognitive distortions were observed more in the clinical group than in the control group, consistent with the literature. Studies on cognitive distortions are mostly related to depression, and studies on anxiety disorders are comparatively limited [13,19]. It was reported that cognitive distortions are more common in individuals with anxiety disorders than in healthy individuals [31,32]. In a study conducted with adolescents diagnosed with major depressive disorder, it was stated that cognitive distortions were higher in those with comorbid anxiety disorders [20]. In addition, although there are studies indicating that cognitive distortions in anxiety disorders in the pre-treatment process are associated with accompanying depression and are independent of the level of anxiety, it has been emphasized in these studies that a significant decrease in cognitive distortion levels was observed after treatment for anxiety [33,34].

All cognitive distortions except “minimizing or disqualifying the positive” were observed more in the GAD group than in the control group. The literature on cognitive distortions observed in GAD emphasizes that patients generally have cognitive distortions related to the meanings attributed to concerns about risks and threats, coping skills and the intolerance of uncertainty [11,12,35]. In another study, it was found that the cognitive distortion of underestimating or ignoring the positive in interpersonal relationships was associated with treatment non-adherence and it was stated that this may be related to excessive focus on the negative consequences of the therapy process, medication efficacy and side effects [36]. The finding that the cognitive distortion of “minimizing or disqualifying the positive” was similar in the GAD and control groups is a finding different from the existing literature. However, it is noteworthy that there are few studies in the literature examining cognitive distortions in GAD. It is clear that more research is needed in this field.

When the cognitive distortions in PD were examined, similar to the literature, cognitive distortions such as catastrophizing and emotional reasoning differed between the control group and the PD group, but differences were also observed in other distortions between the control group and the PD group such as mindreading, all-or-nothing thinking and should statements, suggesting that PD therapy processes should not be limited only to certain distortions. Studies emphasized that in addition to cognitive processes such as low self-efficacy and hypersensitivity to anxiety, cognitive distortions such as catastrophizing and emotional reasoning were predominantly present. This situation made the process chronic, along with dysfunctional behaviors, the need to focus especially on these cognitive distortions in therapy and that recovery is parallel with the improvement of these cognitive distortions [37,38,39]. In a prospective psychotherapy study on the changes in cognitions after focusing on cognitive distortions in individuals diagnosed with PD, it was identified that significant improvements were observed in PD severity and anxiety levels of individuals and this improvement continued at the end of a 1-year follow-up session [39]. In another study, it was reported that cognitive distortions in PD were not related to anxiety levels and were rather associated with a comorbid depressive disorder or depressive symptoms, but it was concluded that a significant decrease was still observed in cognitive distortions after PD treatment [34].

The SAD group differed from the control group in terms of all cognitive distortions. However, in the correlation analysis of the level of anxiety, excluding the effect of the BDI, it was concluded that cognitive distortions did not show a significant relationship with the severity of anxiety in SAD. This suggests that cognitive distortions in SAD may be related to the severity of depressive symptoms rather than the severity of anxiety. Research studies on cognitive distortions in individuals diagnosed with SAD are limited and their results are contradictory. Most of these studies compared individuals with depression or healthy individuals [14,40,41,42]. One study concluded that cognitions related to negative self-perception increased social anxiety [40]. Another survey concluded that negative cognitions related to social situations observed in SAD were not related to depression, that they were cognitions specific to SAD and that this situation distinguished individuals diagnosed with SAD from individuals diagnosed with depression [41]. A further study reported that cognitive distortions were more related to depression and trait anxiety in individuals diagnosed with SAD [13].

In the present study, the SAD group had significantly higher scores than the PD group in terms of “labeling”, “personalization” and “minimizing or disqualifying the positive” cognitive distortions, and the GAD group had significantly higher scores than the PD group in terms of the “mental filter” cognitive distortion. The SAD and GAD groups were similar in terms of all the cognitive distortions. Although there are studies examining cognitive distortions in anxiety disorders in the literature, no comparative study was found that examined anxiety disorders in terms of cognitive distortions. In addition, studies examining cognitive distortions in anxiety generally evaluated cognitive distortions without a specific anxiety diagnosis. The number of studies examining and interpreting each type of cognitive distortion individually is quite limited and the results differ from each other, as do this study’s results. In one study, catastrophizing, overgeneralization and mental filtering [43], in a second study, overgeneralization [44] and in another study, underestimation of coping skills and mindreading cognitive distortions [45] were found to be more predictive of anxiety. The fact that the PD, SAD and GAD groups each showed almost all of the cognitive distortions more than healthy individuals is a consistent finding with the literature [13,37,46], and the evaluation of cognitive distortions specific to themselves is a subject that has not yet been sufficiently studied. Therefore, it is a significant finding in terms of guiding therapy goals and leading new research.

Although the cognitive distortion scores demonstrated a statistically significant difference between the patient groups and the control group, the fact that the scores of the control group were also relatively high suggested that individuals should be evaluated for cognitive distortions at the subclinical level, considering that people without a diagnosis may also exhibit cognitive distortions.

The severity of depression was higher in the clinical group than in the control group. Anxiety and depression are often interrelated. It is known that depressive symptom severity is often higher in anxiety disorders and major depression is the most common comorbid mental disorder in anxiety disorders [47].

However, the severity of dysfunctional attitudes in the clinical group was generally higher than in the control group. The severity of dysfunctional attitudes was higher in the SAD group than in both the GAD and PD groups. Studies have revealed a positive relationship between anxiety and dysfunctional attitudes [48,49,50,51,52]. This relationship may be related to the negative effects of dysfunctional attitudes on interpersonal relationships, problem-solving and coping skills, which may lead to increased and sustained anxiety and stress [53].

Dysfunctional attitudes showed a positive correlation with cognitive distortions in both individuals with SAD, GAD and PD in this study. There is a strong relationship between dysfunctional attitudes and cognitive distortions. Likewise, in a study conducted in individuals diagnosed with depression, it was reported that cognitive distortions were associated with automatic thoughts and dysfunctional attitudes rather than depressive symptoms [25]. In the current literature, we did not find a study comparing PD, SAD and GAD in terms of dysfunctional attitudes. Further research is needed in this area.

This study has a number of limitations and requires caution in the interpretation of its results. The cross-sectional nature of this study leads to the inability to interpret the data obtained as causal. The small sample size reduces the statistical power of the data. In addition, the fact that the groups differed in terms of sociodemographic characteristics that are likely to affect cognitive distortions, such as gender and the presence of a history of suicide attempts, is among the limitations of our study.

This study is a pioneering study in which anxiety disorders were evaluated and compared in terms of cognitive distortions. Research on cognitive distortions is predominantly related to depression, and studies on anxiety disorders are relatively limited. There are no comparative studies examining anxiety disorders such as PD, GAD and SAD in terms of cognitive distortions. Considering the scarcity of studies evaluating cognitive distortions in anxiety disorders in the literature, this study is significant in terms of guiding therapy goals and future studies. According to the cognitive model, cognitive distortions have a significant impact on the emergence of symptoms and the chronicity of the process in anxiety disorders. Studies on whether there are cognitive distortions specific to PD, GAD and SAD differ from each other. Knowing the existence of specific cognitive distortions for PD, GAD and SAD will guide clinicians during the treatment process. Although there are currently many treatment options with recognized efficacy in the treatment of anxiety disorders, many patients do not achieve complete remission and still experience residual symptoms. This indicates the importance of focusing on cognitive distortions in more detail in the therapy process.

## 5. Conclusions

Cognitive distortions in anxiety disorders are one of the main goals of cognitive behavioral therapy. At the same time, anxiety disorders are mental disorders with high comorbidity. The differences in cognitive distortions observed in anxiety disorders emphasize the importance of a primary diagnosis. If the primary diagnosis is not considered, it becomes harder to focus on the cognitive distortions specific to diagnosis. However, the importance of common cognitive distortions in anxiety disorders should not be ignored. Therapists should keep in mind that, after addressing the basic cognitive distortions specific to the diagnosis, other common cognitive distortions may also need intervention.

## Figures and Tables

**Table 1:** Sociodemographic variables of clinical and healthy groups.

	PD N (%)	GAD N (%)	SAD N (%)	Control N (%)	Test Statistic	*p*
**Sex**						
Female	30 (60.0)	32 (64.0)	19 (38.0)	50 (54.9)	𝜒^2^ = 7.920	0.049 *
Male	20 (40.0)	18 (36.0)	31 (62.0)	41 (45.1)		
**Marital Status**						
Married	23 (46.0)	19 (38.0)	16 (32.0)	33 (36.2)	𝜒^2^ = 2.238	0.517 *
Single	27 (54.0)	31 (62.0)	34 (68.0)	58 (63.8)		
**Comorbid Medical Illness**						
Yes	10 (20.0)	8 (16.0)	13 (26.0)	15 (16.5)	𝜒^2^ = 2.282	0.528 *
No	40 (80.0)	42 (84.0)	37 (74.0)	76 (83.5)		
**Presence of Mental Disorder in the Family**						
Yes	18 (36.0)	9 (18.0)	16 (32.0)	9 (9.9)	𝜒^2^ = 17.080	0.001 *
No	32 (64.0)	41 (82.0)	34 (68.0)	82 (90.1)		
**Smoking**						
Yes	19 (38.0)	25 (50.0)	17 (34.0)	31 (34.1)	𝜒^2^ = 3.983	0.278 *
No	31 (62.0)	25 (50.0)	33 (68.0)	60 (65.9)		
**Alcohol Use**						
Yes	7 (14.0)	5 (10.0)	10 (20.0)	20 (21.9)	𝜒^2^ = 3.864	0.280 *
No	43 (86.0)	45 (90.0)	40 (80.0)	71 (78.1)		
**Suicide Attempt History**						
Yes	3 (6.0)	7 (14.0)	3 (6.0)	1 (1.1)	𝜒^2^ = 9.827	0.019 *
No	47 (94.0)	43 (86.0)	47 (94.0)	90 (98.9)		
	**PD** **(M ± SD)**	**GAD** **(M ± SD)**	**SAD** **(M ± SD)**	**Control** **(M ± SD)**	**F**	***p***
**Age**	29.10 ± 8.16	28.62 ± 8.18	26.92 ± 8.18	27.67 ± 6.75	0.838	0.474 **
**Duration of Education (Year)**	12.64 ± 3.02	11.28 ± 3.31	11.50 ± 2.80	12.56 ± 3.92	2.448	0.064 **

PD: panic disorder; GAD: generalized anxiety disorder; SAD: social anxiety disorder, N = number of cases, M = mean, SD = standard deviation, * Pearson chi-square test, ** one-way ANOVA Test, 𝜒^2^: chi-square test statistic.

**Table 2:** Comparison of clinical scale scores between groups.

		PD (M ± SD)	GAD (M ± SD)	SAD (M ± SD)	Control (M ± SD)	F	*p*
BDI		19.94 ± 10.75	24.18 ± 11.63	25.12 ± 10.88	7.11 ± 4.53	60.28	<0.001 *
DAS	Total Score	132.46 ± 33.65	144.02 ± 28.78	162.46 ± 38.16	118.98 ± 27.87	21.71	<0.001 *
	P	28.74 ± 12.47	33.28 ± 12.51	41.20 ± 12.77	28.45 ±10.07	14.59	<0.001 *
	D	24.34 ± 7.94	25.54 ± 7.51	28.46 ± 8.00	20.13 ± 5.90	16.13	<0.001 *
STAI	State	46.92 ± 11.24	49.66 ± 10.53	40.32 ± 5.73	30.55 ± 5.59	74.83	<0.001 *
	Trait	52.16 ± 8.67	55.70 ± 7.77	51.30 ± 6.07	35.05 ± 7.41	105.10	<0.001 *
CDS	Total Score	73.16 ± 24.03	83.02 ± 25.84	86.76 ± 25.47	57.93 ± 23.87	19.20	<0.001 *
	IP	37.58 ± 11.83	42.70 ± 13.86	43.76 ± 13.16	29.57 ± 11.89	18.86	<0.001 *
	PA	35.58 ± 13.26	40.32 ± 14.14	43.00 ± 13.01	28.36 ± 12.34	16.83	<0.001 *

PD: panic disorder; GAD: generalized anxiety disorder; SAD: social anxiety disorder; CDS: Cognitive Distortions Scale; IP: interpersonal; PA: personal achievement; BDI: Beck Depression Inventory; DAS: Dysfunctional Attitude Scale; P: perfectionism/performance evaluation; D: dependency; STAI: State-Trait Anxiety Inventory, SD: SD = standard deviation, * one-way ANOVA test.

**Table 3:** Post hoc comparisons of clinical scale scores between groups.

		Post Hoc Tests (Adjusted *p*-Value)					
		PD-GAD	PD- SAD	GAD-SAD	PD-Control	GAD-Control	SAD-Control
BDI		0.130	**0.031**	1.000	**<0.001**	**<0.001**	**<0.001**
DAS	Total Score	0.414	**<0.001**	**0.023**	0.098	**<0.001**	**<0.001**
	P	0.321	**<0.001**	**0.005**	1.000	0.119	**<0.001**
	D	1.000	**0.026**	0.254	**0.006**	**<0.001**	**<0.001**
STAI	State	0.583	**<0.001**	**<0.001**	**<0.001**	**<0.001**	**<0.001**
	Trait	0.139	1.000	**0.029**	**<0.001**	**<0.001**	**<0.001**
CDS	Total Score	0.280	**0.038**	0.977	**0.003**	**<0.001**	**<0.001**
	IP	0.257	0.088	1.000	**0.002**	**<0.001**	**<0.001**
	PA	0.425	**0.029**	1.000	**0.011**	**<0.001**	**<0.001**

PD: panic disorder; GAD: generalized anxiety disorder; SAD: social anxiety disorder; CDS: Cognitive Distortions Scale; IP: interpersonal; PA: personal achievement; BDI: Beck Depression Inventory; DAS: Dysfunctional Attitude Scale; P: perfectionism/performance evaluation; D: dependency; STAI: State-Trait Anxiety Inventory.

**Table 4:** Comparison of cognitive distortions between groups.

		PD (Mean Rank)	GAD (Mean Rank)	SAD (Mean Rank)	Control (Mean Rank)	df	*X^2^*	*p*
Mindreading	IP	146.24	135.00	128.64	95.24	3	22.20	<0.001 *
	PA	129.62	139.74	136.27	97.58	3	17.55	0.001 *
Catastrophizing	IP	130.46	149.22	134.39	92.94	3	26.34	<0.001 *
	PA	127.82	140.09	150.73	90.43	3	31.59	<0.001 *
All-or-Nothing Thinking	IP	131.96	149.22	134.39	92.45	3	25.89	<0.001 *
	PA	125.39	144.38	137.90	96.46	3	20.56	<0.001 *
Emotional Reasoning	IP	120.68	135.59	151.67	92.45	3	23.90	<0.001 *
	PA	131.89	135.35	153.40	89.33	3	33.68	<0.001 *
Labeling	IP	111.31	136.87	161.64	95.27	3	33.88	<0.001 *
	PA	109.83	140.71	163.59	92.91	3	39.93	<0.001 *
Mental Filter	IP	108.23	159.28	136.45	98.49	3	29.36	<0.001 *
	PA	109.54	152.71	139.03	99.97	3	23.88	<0.001 *
Overgeneralization	IP	122.00	155.23	143.66	89.19	3	37.13	<0.001 *
	PA	114.64	147.15	147.77	95.42	3	27.84	<0.001 *
Personalization	IP	110.07	143.87	156.24	95.08	3	32.82	<0.001 *
	PA	112.14	141.39	151.70	97.80	3	25.68	<0.001 *
Should Statements	IP	136.99	135.67	147.63	89.52	3	31.64	<0.001 *
	PA	134.40	128.22	148.54	94.54	3	23.95	<0.001 *
Minimizing or Disqualifying the Positive	IP	116.12	129.26	156.94	99.40	3	23.80	<0.001 *
	PA	120.11	122.80	154.86	101.90	3	19.36	<0.001 *

PD: panic disorder; GAD: generalized anxiety disorder; SAD: social anxiety disorder; IP: interpersonal; PA: personal achievement; df: degree of freedom, * Kruskal–Wallis H Test.

**Table 5:** Post hoc comparisons of cognitive distortions between groups.

		Post Hoc Tests (Adjusted *p*-Value)					
		PD-GAD	PD- SAD	GAD-SAD	PD-Control	GAD-Control	SAD-Control
Mindreading	IP	1.000 *	1.000 *	1.000 *	<0.001 *	0.006 *	0.035 *
	PA	1.000 *	1.000 *	1.000 *	0.048 *	0.003 *	0.008 *
Catastrophizing	IP	1.000 *	1.000 *	1.000 *	0.012 *	<0.001 *	0.004 *
	PA	1.000 *	0.557 *	1.000 *	0.012 *	<0.001 *	<0.001 *
All-or-Nothing Thinking	IP	1.000 *	1.000 *	1.000 *	0.007 *	<0.001 *	0.001 *
	PA	1.000 *	1.000 *	1.000 *	0.102 *	<0.001 *	0.004 *
Emotional Reasoning	IP	1.000 *	0.146 *	1.000 *	0.265 *	0.007 *	<0.001 *
	PA	1.000 *	0.712 *	1.000 *	0.003 *	0.001 *	<0.001 *
Labeling	IP	0.378 *	0.002 *	0.429 *	1.000 *	0.004 *	<0.001 *
	PA	0.147 *	0.001 *	0.574 *	0.969 *	<0.000 *	<0.000 *
Mental Filter	IP	0.001 *	0.244 *	0.586 *	1.000 *	<0.001 *	0.011 *
	PA	0.010 *	0.194 *	1.000 *	1.000 *	<0.000 *	0.008 *
Overgeneralization	IP	0.096 *	0.697 *	1.000 *	0.041 *	<0.001 *	<0.001 *
	PA	0.108 *	0.096 *	1.000 *	0.674 *	<0.001 *	<0.001 *
Personalization	IP	0.084 *	0.005 *	1.000 *	1.000 *	<0.001 *	<0.001 *
	PA	0.198 *	0.024 *	1.000 *	1.000 *	0.002 *	<0.000 *
Should Statements	IP	1.000 *	1.000 *	1.000 *	0.001 *	0.001 *	<0.001 *
	PA	1.000 *	1.000 *	0.837 *	0.006 *	0.032 *	<0.000 *
Minimizing or Disqualifying the Positive	IP	1.000 *	0.017 *	0.260 *	0.992 *	0.080 *	<0.001 *
	PA	1.000 *	0.067 *	0.115 *	0.784 *	0.497 *	<0.000 *

PD: panic disorder; GAD: generalized anxiety disorder; SAD: social anxiety disorder; IP: interpersonal; PA: personal achievement; df: degree of freedom, * Mann–Whitney U Test.

**Table 6:** Correlations of cognitive distortions with STAI and DAS in BDI control in clinical groups.

		Control for	STAI		DAS		
			State	Trait	Total	P	D
PD	CDS-Total	None	0.411 **	0.555 **	0.638 **	0.644 **	0.495 **
		BDI	0.225	0.380 **	0.596 **	0.604 **	0.450 **
	CDS-IP	None	0.500 **	0.605 **	0.580 **	0.565 **	0.411 **
		BDI	0.336 *	0.444 **	0.528 **	0.512 **	0.352 *
	CDS-PA	None	0.298 *	0.467 **	0.639 **	0.664 **	0.531 **
		BDI	0.107	0.288 *	0.598 **	0.627 **	0.492 **
GAD	CDS-Total	None	0.544 **	0.568 **	0.468 **	0.571 **	0.434 **
		BDI	0.349 *	0.380 **	0.351 *	0.504 **	0.314 *
	CDS-IP	None	0.546 **	0.504 **	0.545 **	0.613 **	0.530 **
		BDI	0.390 **	0.321 *	0.456 **	0.557 **	0.442 **
	CDS-PA	None	0.459 **	0.545 **	0.321 *	0.443 **	0.273
		BDI	0.242	0.366 **	0.180	0.355 *	0.127
SAD	CDS-Total	None	−0.098	0.340 *	0.650 **	0.640 **	0.359 *
		BDI	−0.067	0.144	0.531 **	0.524 **	0.263
	CDS-IP	None	−0.086	0.306 *	0.651 **	0.639 **	0.384 **
		BDI	−0.052	0.104	0.534 **	0.523 **	0.298 *
	CDS-PA	None	−0.105	0.357 *	0.614 **	0.608 **	0.314 *
		BDI	−0.076	0.172	0.479 **	0.477 **	0.203

PD: panic disorder; GAD: generalized anxiety disorder; SAD: social anxiety disorder; CDS: Cognitive Distortions Scale; IP: interpersonal; PA: personal achievement; BDI: Beck Depression Inventory; DAS: Dysfunctional Attitude Scale; P: perfectionism/performance evaluation; D: dependency; STAI: State-Trait Anxiety Inventory, * *p* < 0.05; ** *p* < 0.01.

## Data Availability

The data that support the findings of this study are available on request from the corresponding author. The data are not publicly available due to privacy or ethical restrictions.
